# Higher plasma AFB1 concentration is associated with increased risk of HPV 16 and HPV 18 detection and persistence among Ugandan women

**DOI:** 10.1186/s12940-025-01197-0

**Published:** 2025-07-07

**Authors:** Yan Tong, Miriam Nakalembe, Collins Mpamani, Carolyn Nakisige, Jane Namugga, Grace Banturaki, Philiph Tonui, Omenge Orang’o, Kapten Muthoka, Anthony Ngeresa, John Groopman, Sean Burke, Aaron Ermel, Beverly Musick, Patrick Loehrer, Darron R. Brown

**Affiliations:** 1 Department of Biostatistics and Health Data Science, Indiana University School of Medicine, Indianapolis, IN 46202 USA; 2 Department of Obstetrics and Gynaecology, Infectious Diseases Institute, Makerere University, Kampala, Uganda; 3 Uganda Cancer Institute, Kampala, Uganda; 4 Department of Reproductive Health, Moi University, Eldoret, Kenya; 5 Department of Obstetrics and Gynecology, Medical College, Aga Khan University, Nairobi, Kenya; 6 Academic Model Providing Access to Healthcare (AMPATH), Eldoret, Kenya; 7 Department of Environmental Health and Engineering, Johns Hopkins Bloomberg School of Public Health, Baltimore, MD 21205 USA; 8 Department of Medicine, Indiana University School of Medicine, Indianapolis, IN USA; 9 Department of Microbiology and Immunology, Indiana University School of Medicine, Indianapolis, IN USA; 10 Melvin and Bren Simon Comprehensive Cancer Center, Indiana University School of Medicine, Indianapolis, IN USA

## Abstract

**Introduction:**

Aflatoxins are environmental hazards; potent carcinogenic and immunosuppressive agents that contaminates corn and other crops. A high proportion of hepatocellular carcinoma cases are caused by exposure to dietary aflatoxins. Cervical cancer is common among Ugandan women; this malignancy is caused by persistent infection with oncogenic HPV types. An analysis was performed to examine associations between plasma aflatoxin B_1_ (AFB_1_) detection and oncogenic HPV detection (HPV types 16, 18, 31, 33, 35, 39, 45, 51, 52, 56, 58, 59, 66, and 68) and persistence among Ugandan women.

**Methods:**

Ugandan women were enrolled in a prospective cohort study. Annual cervical swabs (Enrollment, Month 12 and Month 24) were tested for oncogenic HPV. Plasma AFB_1_ concentration was measured (as AFB_1_-lysine conjugate, or AFB_1_-lys) at Enrollment and Month 12. Multivariable regression models were fitted to examine associations of plasma AFB_1_-lys concentrations and oncogenic HPV controlling for demographic and behavioral characteristics.

**Results:**

The analytical sample consisted of 114 women with a mean age of 33.2 years; 60 women were living with HIV; 59 were receiving antiretroviral therapy (ART) at enrollment. AFB_1_-lysine adducts (AFB_1_-lys) was detected in plasma from all 114 women. Multivariable regression models showed that plasma AFB_1_-lys concentration was associated with a higher risk of detection of HPV 16 (OR = 2.64, 95% CI = 1.42–4.90, *p* = 0.002) and HPV 18 (OR = 2.24, 95% CI = 1.27–3.96, *p* = 0.005), and persistence of HPV 16 (OR = 3.16, 95% CI = 1.59–6.26, *p* = 0.001) and HPV 18 (OR = 2.06, 95% CI = 1.09–3.90, *p* = 0.025), controlling for age, marital status, years of education, home ownership, distance to health care, number of lifetime sex partners, age of first sex, and HIV status.

**Conclusions:**

AFB_1_ is an environmental hazard that is prevalent among Ugandan women. Higher plasma AFB_1_-lys concentration was associated with detection and persistence of HPV 16 and HPV 18; this association was independent of HIV status. As a result, these women may be at increased risk of cervical cancer. Further studies are needed to determine the mechanisms involved.

## Introduction

Cervical cancer is the fourth most common cancer among women globally and is responsible for nearly 300,000 deaths annually; 90% of these deaths occur among women living in low- and middle-income countries [1, 2, 3, 4, 5]. The highest age-standardized incidence rates worldwide for cervical cancer occur in sub-Saharan Africa; this malignancy is the leading cause of cancer-related deaths in women in this region [5, 6, 7, 8]. The age-standardized incidence and mortality rates of cervical cancer in Ugandan women are 56.2 and 41.4 per 100,000 cases per year [4], in contrast to 4 and 1 per 100,000 cases per year in women living in the U.S [9, 10]. Oncogenic types of human papillomaviruses (also known as “high-risk”, or HR-HPV) are the causative agents of cervical cancer [11, 12, 13]. Persistence of HR-HPV is a key determining factor for development of this malignancy [14, 15]. HPV 16 and HPV 18 are two HR-HPV types that cause approximately 72% of cervical cancers in sub-Saharan Africa and nearly 80% of cervical cancers worldwide [13]. Although only a small percentage of HPV-infected women will eventually develop cervical cancer, (an estimated 0.5 to 1% in the United States), women with persistent detection of HR-HPV are at the greatest risk for this malignancy [14, 15, 16]. Women who do not undergo regular screening, and those who are HIV-infected are at higher risk of this malignancy [17]. Determining the modifiable factors associated with persistent HR-HPV infection is important for reducing the cancer risk in women living in these regions.

Dietary aflatoxins, including aflatoxin B_1_ (AFB_1_) may be such a risk factor for cervical cancer. Aflatoxins are a group of mycotoxins produced by certain strains of Aspergillus [18, 19, 20, 21]. Aspergillus contamination of beans, corn, sorghum, peanuts and other crops occurs during growth or after harvesting in Uganda and other sub-Saharan African countries, and other areas of the world, especially in warm and humid environments [21, 22, 23]. Over 90% of segments of the Ugandan general population have been found with aflatoxins in plasma [24]. Aflatoxins are causative agents of liver cancer and may be associated with other cancers [25, 26, 27, 28]. Aflatoxins are classified by the International Agency for Research on Cancer (IARC) as class I carcinogens [29]. In addition to causing cancer, aflatoxins are potent immunosuppressive agents [30, 31, 32, 33]. AFB1-lysine (AFB1-lys) is an adduct that can be detected in plasma and is validated indicator of long term AFB1 exposure [34]. We previously showed that plasma AFB_1_-lys biomarkers are associated with HR-HPV detection and persistence among HIV-uninfected Kenyan women enrolled in a prospective study of HPV epidemiology [35]. Plasma AFB_1_-lys was detected in 57% of women and was associated with detection of HR-HPV in cervical samples based on a cross-sectional analysis of the study enrollment data [35]. An additional longitudinal analysis of these women furtherly demonstrated an association between plasma AFB_1_-lys detection and persistence of HR-HPV [36].

The current analysis was performed to (1) determine if women living in another sub-Saharan country, Uganda, are exposed to AFB_1_, (2) determine if higher plasma AFB_1_-lys detection and concentration are associated with detection and persistence of HR-HPV in Ugandan women including, and (3) determine if the associations of plasma AFB_1_-lys concentrations with HR-HPV detection and persistence differ between women living with HIV (WLWH) and women who were not HIV-infected.

## Methods

### Study population

Ugandan women were enrolled at the Uganda Cancer Institute (UCI)–Kawempe Division, Mulago, Uganda from 2020 to 2021. These women were participants in the East African Consortium for Human Papillomavirus and Cervical Cancer in Women Living with HIV/AIDS [37]. This study was a prospective cohort study investigating biological, behavioral, and environmental risk factors for HR-HPV infection and persistence. A balanced cohort of Ugandan women was recruited at a 1:1 ratio of WLWH and a control group of women not living with HIV (WNLWH), as previously described [37]. Briefly, women aged 18 to 60 years living within 30 km of Kampala presenting for screening at the Uganda Cancer Institute (UCI) were asked to participate in the study if they had a normal visual inspection with acetic acid (VIA).

A total of 120 Ugandan women (61 WLWH and 59 WNLWH) consented to participation and enrolled in the study; of all enrolled participants, 114 women completed Month 12 and Month 24 follow-up visits, including 60 WLWH and 54 WNLWH, who represented the analytical cohort for this analysis. For all 114 women, plasma was obtained at Enrollment and the Month 12 visit, and at least two adequate cervical swab samples (based on beta globin testing) were obtained at Enrollment, the Month 12 visit, or the Month 24 visit.

### Collection of women’s data

At study enrollment, trained researchers conducted structured face-to-face interviews with participants to capture social, behavioral, and biological information, including age, marital status, educational level, home ownership, travel time to the local clinic, number of lifetime sexual partners, and age of first sex [38]. At each study visit, a cervical swab for HPV testing was collected by a nurse or physician as part of the cervical inspection for cancer screening. Swabs were immediately placed in standard transport media and frozen at -20 °C. Swabs were delivered in batches to the UCI Laboratory (Kampala). Plasma was collected and frozen at -20 °C, then sent to the Johns Hopkins University for AFB_1_ testing.

### HPV testing

Cervical specimens were analyzed using the Roche Cobas Assay (Roche Molecular Systems, Inc., Branchburg, NJ USA) to determine the presence of HR-HPV types. This assay detects 14 h-HPV types as designated by the International Agency for the Research on Cancer (IARC) [39], including HPV 16, HPV 18, and 12 additional HR-HPV types that are detected as a group (HPV types 31, 33, 35, 39, 45, 51, 52, 56, 58, 59, 66, and 68). HPV 16-positive, HPV-negative, and human beta-globin (used to assess specimen adequacy) controls provided by the manufacturer were tested with each batch of samples.

### AFB_1_-lysine adduct (AFB_1_-lys) detection in plasma samples

Plasma AFB_1_-lys was measured at the Department of Environmental Health and Engineering of the Johns Hopkins Bloomberg School of Public Health, using a minor variation of the method reported by McCoy and colleagues [40]. The quantitative measurement of the AFB_1_-lys adduct from serum albumin has been rigorously validated. The isotopically-labeled internal standard controls for recovery of the AFB_1_-lys adduct from human samples and this internal standard has been validated [34]. This standard is added to every sample being measured. Every group of samples was tested in duplicate and included a range of standards to provide calibration of the adduct measurements [41, 42, 43]. Cross validation experiments have been performed to compare results using our mass spectrometry method with determinations using fluorescence HPLC detection and ELISA techniques that has further validated the mass spectrometry technology [40]. This normalization strategy provides justification for comparing samples across different investigations [44, 45]. Briefly, plasma (150 µL) was spiked with an internal standard (0.5 ng AFB_1_-d4-lysine in 100 µL), combined with Pronase (EMD Millipore, Billerica MA, USA) protease solution (3.25 mg in 0.5 mL phosphate-buffered saline), and incubated for 18 h at 37 °C. Solid-phase extraction–processed samples (Oasis MAX columns; Waters, Milford, MA, USA) were analyzed with ultra-high pressure liquid chromatography (UHPLC)-isotope dilution mass spectrometry on a ThermoFisher Scientific (San Jose, CA, USA) system composed of a Vanquish UHPLC and a TSQ Quantis triple quadrupole mass spectrometer in positive electrospray ionization mode [41, 46].

### Persistent HPV 16 or HPV 18 detection

HPV 16 and HPV 18 testing results obtained from the Enrollment, Month 12 and Month 24 cervical samples were combined to determine the type-specific HPV detection category for each woman. To be included in the analysis, at least two of the three participant’s cervical samples (Enrollment, Month 12, or Month 24) had to be available and valid; one of the three samples could be missing. The type-specific HPV detection categories for HPV 16 and HPV 18 were defined as follows: (I) *No detection*: No detection for the specific HPV type in any of the three samples; (II) *Incident detection*: One sample was positive for detection of a specific HPV type, but other samples were negative for that type; (III) *Persistent detection*: Two or three samples were positive for detection of a specific HPV type.

### Statistical analysis

For each participant, the value of plasma AFB_1_-lys concentration was calculated as the average of the Enrollment and Month 12 AFB_1_-lys concentration measurements. This AFB_1_-lys concentration value of all participants was ranked and grouped into either a “lower half” category (i.e., below the overall median) or an “upper half” (i.e., above the overall median) category. Demographic and behavioral characteristics of participants at the enrollment (age, marital status, years of education, home ownership, travel time to health care > 30 min, number of lifetime sex partners, age of first sex, and HIV status) were summarized by descriptive statistics and compared between women in the AFB_1_-lys concentration lower half category and upper half category, using Student’s t-tests, Chi-square tests, or Wilcoxon rank sum tests, as appropriate and as indicated in the notes for each Table. In addition, HIV parameters at enrollment (detectable HIV viral load, CD4 cell count, and receiving ART) among WLWH were summarized and compared between those in the AFB_1_-lys concentration lower half category and those in the upper half category using Fisher’s exact test or t-test as appropriate. Detections of HPV 16, HPV 18, Non16/18 HR-HPV, or any HR-HPV at each study visit (Enrollment, Month 12, or Month 24) were summarized and compared between women in the AFB_1_-lys concentration lower half and the upper half categories using Fisher’s exact tests or Chi-square tests as appropriate. Generalized estimating equation (GEE) logistic regression models were fitted to examine associations of HPV detection with plasma AFB_1_-lys concentration, controlling for demographic and behavioral characteristics and HIV status as confounders. Furthermore, frequencies and percentages of HPV 16 and HPV 18 detections (“No detection”, “Incident detection” and “Persistent detection”) among women were compared between those in the AFB_1_-lys concentration lower half category and those in the upper half category using Fisher’s exact tests. Plasma AFB_1_-lys concentrations were summarized in mean, standard deviation (std), median and interquartile range (IQR) and compared among women with different HPV detection status using Wilcoxon rank sum tests. Ordinal logistic regression models were fitted to examine associations of HPV 16 and HPV 18 detection (persistent detection vs. incident detection vs. no detection) with plasma AFB_1_-lys concentration controlling for demographic and behavioral characteristics and HIV status as covariates. The proportional odds assumption was examined for each fitted ordinal logistic regression model to ensure validity of the model. All analyses were performed using SAS Version 9.4 (Cary, NC).

### Ethics considerations

Study approval was granted from the local review board at the Uganda Cancer Institute (UCI)–Kawempe Division, Mulago, Uganda and the Institutional Review Board of Indiana University. The study was explained to all participants, and all signed informed consent for participation.

## Results

### Overall characteristics of participants and plasma AFB_1_-lys concentration

Table 1 shows a summary of the demographics, behavioral and environmental factors among 114 Ugandan women at the study enrollment. All women had AFB_1_-lys detected in plasma samples at both the Enrollment and Month 12 visits. The mean plasma AFB_1_-lys concentration for all 114 women was 1.257 pg/µL (standard deviation: 0.977); the range was 0.200 to 4.812 pg/µL. Based on the median AFB_1_-lys concentration value of 0.8790 pg/µL, participants were grouped into a “lower half” or “upper half” AFB_1_-lys concentration category with 57 participants in each category. The mean (range) AFB_1_-lys concentration in the lower half category was 0.584 (0.200 to 0.876) pg/µL, and 1.930 (0.882 to 4.812) pg/µL in the upper half category.

**Table 1:** Summary of demographics and behavioral factors at the enrollment among 114 Uganda women by averaged plasma aflatoxin concentration category

Variable	Plasma aflatoxin concentration category^1^			*p*-value
	Overall (*N* = 114) Mean 1.257 pg/uL	Lower half (*N* = 57) Mean 0.584 pg/uL	Upper half (*N* = 57) Mean 1.930 pg/uL	
Age (years), median (IQR)	33.2 (28.2, 39.1)	33.3 (28.8, 39.0)	32.2 (28.1, 39.5)	0.829^2^
Years of education, median (IQR)	9.0 (7.0, 13.0)	9.0 (7.0, 12.0)	9.0 (6.0, 13.0)	0.881^2^
Married or living with a partner, n (%)	64 (56.6%)	34 (60.7%)	30 (52.6%)	0.386^3^
Home ownership, n (%)	24 (21.1%)	11 (19.3%)	13 (22.8%)	0.646^3^
Travel to health clinic > 30 min, n (%)	46 (40.4%)	26 (45.6%)	20 (35.1%)	0.252^3^
Life-time sex partners, median (IQR)	3.0 (2.0, 5.0)	3.0 (2.0, 5.0)	3.0 (2.0, 5.0)	0.573^4^
Age of sex debut, median (IQR)	17.0 (16.0, 19.0)	17.0 (15.0, 19.0)	18.0 (16.0, 19.0)	0.419^2^
HIV-infected, n (%)	60 (52.6%)	32 (56.1%)	28 (49.1%)	0.453^3^

^1^ The range of the plasma aflatoxin concentration was 0.200 to 4.812 pg/uL for all women, 0.200 to 0.876 pg/uL for those in the lower half aflatoxin concentration category, and 0.882 to 4.812 pg/uL for those in the upper half aflatoxin concentration category

^2^ p-value obtained from Student’s t-test

^3^ p-value obtained from Chi-square test

^4^ p-value obtained from Wilcoxon rank sum test

The average age of all 114 women was 33.2 years (IQR 28.2, 39.1). At enrollment there were no differences in age, years of education, marital status (e.g., being married or living with a partner), home ownership, travel time to health care, number of lifetime sex partners, or age of first sex between women in the AFB_1_-lys concentration lower half category and those in the upper half category (Table 1).

### HIV parameters among 60 WLWH by plasma AFB_1_-lys category

There were 60 WLWH included in this analysis. Of the 60 WLWH, 32 were grouped into the AFB_1_-lys concentration lower half category (mean 0.565 pg/µL), and 28 in the upper half category (mean 2.043 pg/µL). A summary of HIV parameters at the study enrollment by AFB_1_-lys concentration category is shown in Table 2. Of the 60 WLWH, 8 (13.3%) had a detectable HIV viral load and 52 (86.7%) had an undetectable HIV viral load. The median CD4 cell count for all 60 WLWH was 822 cells/µL. Fifty-nine of 60 WLWH (98.3%) were receiving ART at the Enrollment visit; one woman began ART after being enrolled in the study. There were no significant differences in HIV parameters between women in the lower half or the upper half AFB_1_-lys concentration category.

**Table 2:** Summary of HIV parameters at the enrollment among 60 WLWH by plasma aflatoxin concentration category

Variable	Plasma aflatoxin concentration category^1^			*p*-value
	Overall (*N* = 60) Mean 1.255 pg/uL	Lower half (*N* = 32) Mean 0.565 pg/uL	Upper half (*N* = 28) Mean 2.043 pg/uL	
Detectable HIV viral load, n (%)	8 (13.3%)	4 (12.5%)	4 (14.3%)	1.000^2^
CD4 + cell count (cells/uL), median (IQR)	822.0 (620.5, 1062)	791.5 (582.0, 986.0)	892.5 (666.5, 1138)	0.223^3^
Receiving ART, n (%)	59 (98.3%)	31 (96.9%)	28 (100.0%)	1.000^2^

^1^ The range of the plasma aflatoxin concentration was 0.200 to 4.812 pg/uL for all women, 0.200 to 0.869 pg/uL for those in the lower half category, and 0.882 to 4.812 pg/uL for those in the upper half category

^2^ p-value obtained from Fisher’s exact test

^3^ p-value obtained from Student’s t-test

### Associations of plasma AFB_1_-lys concentration with HPV detection

Table 3 shows a summary of HPV detection at the Enrollment, Month 12, and Month 24 visits among all 114 women by the AFB_1_-lys concentrations category. In general, women with plasma AFB_1_-lys concentrations in the upper half category were more likely to have HPV 16, HPV 18, Non16/18 HR-HPV, and HR-HPV detected at all three time points, compared to women with AFB_1_-lys concentrations in the lower half category. Significant differences in Non-16/18 HR-HPV (12.3% vs. 29.8%, *p* = 0.022) and any HR-HPV (14.0% vs. 33.3%, *p* = 0.015) were found between women in the lower half and the upper half AFB_1_-lys concentration categories at the Month 24 visit. Women with plasma AFB_1_-lys concentration in the upper half category were more likely to have either HPV 16 or HPV 18 detected than women in the lower half category. However, due the small number of cases, differences in HPV 16 and HPV 18 detections were not significant between the plasma AFB_1_-lys concentration categories (upper half vs. lower half).

**Table 3:** Summary of HPV detection at enrollment, month 12 and month 24 among 114 women by plasma aflatoxin concentration category

HPV detection	Visit	Plasma aflatoxin concentration category^1^			p-value
		Overall *N* = 114	Lower half *N* = 57	Upper half *N* = 57	
HPV 16	Enrollment	7 (6.1%)	1 (1.8%)	6 (10.5%)	0.113^4^
	Month 12	2 (1.8%)	1 (1.8%)	1 (1.8%)	1.000^4^
	Month 24	3 (2.6%)	1 (1.8%)	2 (3.5%)	1.000^4^
HPV 18	Enrollment	8 (7.0%)	3 (5.3%)	5 (8.8%)	0.716^4^
	Month 12	5 (4.4%)	2 (3.5%)	3 (5.4%)	0.679^4^
	Month 24	3 (2.6%)	0	3 (5.3%)	0.243^4^
Non16/18 HR-HPV^2^	Enrollment	49 (43.0%)	21 (36.8%)	28 (49.1%)	0.185^5^
	Month 12	35 (31.0%)	14 (24.6%)	21 (37.5%)	0.137^5^
	Month 24	24 (21.1%)	7 (12.3%)	17 (29.8%)	0.022^5^
HR-HPV^3^	Enrollment	53 (46.5%)	23 (40.4%)	30 (52.6%)	0.189^5^
	Month 12	39 (34.5%)	17 (29.8%)	22 (39.3%)	0.290^5^
	Month 24	27 (23.7%)	8 (14.0%)	19 (33.3%)	0.015^5^

^1^ The mean (range) of the plasma aflatoxin concentration was 1.257 (0.200–4.812) pg/uL for all women, 0.584 (0.200–0.876) pg/uL for those in the lower half category, and

1.930 (0.882–4.812) pg/uL for those in the upper half category

^2^ Non16/18 HR-HPV: HPV 31, 33, 35, 39, 45, 51, 52, 56, 58, 59, 66, 68

^3^ HR-HPV: HPV 16, 18, 31, 33, 35, 39, 45, 51, 52, 56, 58, 59, 66, 68

^4^ p-value obtained from Fisher’s exact test

^5^ p-value obtained from Chi-square test

Table 4 shows the multivariable logistic regression models of HPV 16, HPV 18, Non-16/18 HR-HPV, and HR-HPV detections with demographic and behavioral factors, HIV status, and plasma AFB_1_-lys concentration (pg/µL) using generalized estimating equation (GEE). The measures of HPV detections for all 114 women at Enrollment, Month 12 and Month 24 visits were included in this analysis. A higher plasma AFB_1_-lys concentration was found to be associated with higher risks of both HPV 16 (Odds Ratio (OR) = 2.64, 95% Confidence Interval (CI) = 1.42–4.90, *p* = 0.002) and HPV 18 (OR = 2.24, 95% CI = 1.27–3.96, *p* = 0.005) detections. An older age was associated with a lower chance of Non-16/18 HR-HPV detection (OR = 0.96, 95% CI = 0.92-1.00, *p* = 0.034). Years of education, marital status, home ownership, travel time to the health clinic, number of lifetime sexual partners, age of sexual debut, and HIV status were not associated with any HPV detections. Additional models were conducted to examine interaction effects of HIV status and plasma AFB_1_-lys concentration on HPV detections, and no significant interaction effects were found. Results of the interaction effect models are not shown here.

**Table 4:** Multivariable logistic regression models of HPV detection with demographic and behavioral factors, HIV status, and plasma aflatoxin concentration using generalized Estimation equation (GEE) based on 114 women

Variable	HPV 16		HPV 18		Non16/18 HR-HPV^1^		HR-HPV^2^	
	OR (95%CI)	*p*-value	OR (95%CI)	*p*-value	OR (95%CI)	*p*-value	OR (95%CI)	*p*-value
Age (years)	1.04 (0.94–1.15)	0.408	1.10 (0.99–1.22)	0.087	0.96 (0.92-1.00)	0.034	0.98 (0.94–1.02)	0.250
Years of education	0.96 (0.81–1.14)	0.632	0.99 (0.85–1.15)	0.863	0.99 (0.91–1.08)	0.827	0.98 (0.90–1.06)	0.565
Married or living with a partner	0.31 (0.08–1.21)	0.092	0.67 (0.17–2.64)	0.571	0.76 (0.42–1.36)	0.350	0.72 (0.40–1.28)	0.260
Home ownership	2.29 (0.35–15.13)	0.391	2.57 (0.29–22.57)	0.394	0.92 (0.44–1.94)	0.835	1.12 (0.55–2.28)	0.749
Travel to health clinic > 30 min	0.44 (0.10–1.84)	0.259	1.63 (0.18–14.52)	0.662	0.89 (0.46–1.74)	0.733	0.83 (0.43–1.59)	0.576
Life-time sex partners	1.06 (0.84–1.34)	0.637	0.99 (0.73–1.34)	0.940	0.95 (0.85–1.06)	0.351	0.95 (0.85–1.07)	0.391
Age of sex debut	1.15 (0.88–1.51)	0.311	0.33 (0.04–2.99)	0.323	1.06 (0.93–1.20)	0.395	1.06 (0.94–1.19)	0.365
HIV-infected	1.87 (0.43–8.09)	0.404	0.95 (0.77–1.17)	0.631	1.11 (0.61–2.04)	0.724	1.05 (0.57–1.94)	0.884
Plasma aflatoxin concentration (pg/uL)	2.64 (1.42–4.90)	0.002	2.24 (1.27–3.96)	0.005	1.17 (0.90–1.54)	0.244	1.24 (0.92–1.69)	0.161

^1^ Non16/18 HR-HPV: HPV 31, 33, 35, 39, 45, 51, 52, 56, 58, 59, 66, 68

^2^ HR-HPV: HPV 16, 18, 31, 33, 35, 39, 45, 51, 52, 56, 58, 59, 66, 68

### Associations of plasma AFB_1_-lys concentration with persistent HPV 16 and HPV 18 detection

Table 5 shows the frequencies and percentages of HPV 16 and HPV 18 detections (“no detection”, “incident detection”, or “persistent detection”) among 114 women in the lower half and upper half categories of plasma AFB_1_-lys concentration, and plasma AFB_1_-lys mean (standard deviation or STD) and median (interquartile range or IQR). For HPV 16, 9 of 114 women (7.9%) had a detection episode, including 6 (5.3%) incident detections and 3 (2.6%) persistent detections. Stratified by the plasma AFB_1_-lys concentration category, there was 1 incident detection and 1 persistent detection among women in the lower half category, and 5 incident detections and 2 persistent detections among women in the upper half category. Plasma AFB_1_-lys concentrations were significantly different between women with “no detection”, “incident detection” or “persistent detection” of HPV 16 (*p* = 0.028). For HPV 18, there were 10 detection episodes in 114 women (8.8%), including 5 (4.4%) incident detections and 5 (4.4%) persistent detections; stratified by the plasma AFB_1_-lys concentration category, HPV 18 was detected in 3 women (1 incident detection and 2 persistent detections) in the lower half category and 7 women (4 incident detections and 3 persistent detections) in the upper half category; difference in HPV 18 detection based on plasma AFB_1_-lys concentration category was not statistically significant. In addition, significant differences were not found in plasma AFB_1_-lys concentrations among women with “no detection”, “incident detection” or “persistent detection” for HPV 18.

**Table 5:** Frequency and percentage of HPV 16 and HPV 18 incident and persistent detections among 114 women by plasma aflatoxin concentration category, and the plasma aflatoxin concentration (pg/uL) mean (standard deviation or STD) and median (interquartile range or IQR)

HPV	HPV detection Category	*n* (%)	Plasma aflatoxin concentration category^1^			Plasma aflatoxin concentration (pg/uL)		
			Lower half (*N* = 57) *n* (%)	Upper half (*N* = 57) *n* (%)	*p*-value^2^	Mean (STD)	Median (IQR)	*p*-value^3^
HPV 16	No detection	105 (92.1%)	55 (96.5%)	50 (87.7%)	0.251	1.156 (0.852)	0.860 (0.595–1.425)	0.028
	Incident detection	6 (5.3%)	1 (1.8%)	5 (8.8%)		2.569 (1.470)	2.726 (1.005–3.634)	
	Persistent detection	3 (2.6%)	1 (1.8%)	2 (3.5%)		2.172 (1.931)	1.977 (0.346–4.194)	
HPV 18	No detection	104 (91.2%)	54 (94.7%)	50 (87.7%)	0.394	1.205 (0.954)	0.868 (0.579–1.458)	0.174
	Incident detection	5 (4.4%)	1 (1.8%)	4 (7.0%)		1.935 (1.015)	1.969 (1.296–2.539)	
	Persistent detection	5 (4.4%)	2 (3.5%)	3 (5.3%)		1.653 (1.283)	1.343 (0.703–2.131)	

^1^ The mean (range) of the plasma aflatoxin concentration was 1.257 (0.200–4.812) pg/uL for all women, 0.584 (0.200–0.876) pg/uL for those in the lower half category, and 1.930 (0.882–4.812) pg/uL for those in the upper half category

^2^ p-value obtained from Fisher’s exact test

^3^ p-value obtained from Wilcoxon rank sum test

Table 6 shows the multivariable ordinal logistic regression models of HPV 16 and HPV 18 detections (persistent detection vs. incident detection vs. no detection) with demographic and behavioral factors, HIV status, and plasma AFB_1_-lys concentration in 114 women. For both HPV 16 and HPV 18 detections, age, years of education, marital status, home ownership, travel time to the health clinic, number of lifetime sexual partners, age of sexual debut, and HIV status were not associated with risk of persistent detection. However, a higher level of plasma AFB_1_-lys concentration was strongly associated with a higher risk of persistent detection of HPV 16 (OR = 3.16, 95% CI = 1.59–6.26, *p* = 0.001) or HPV 18 (OR = 2.06, 95% CI = 1.09–3.90, *p* = 0.025). The proportional odds assumption was validated for both ordinal logistic regression models reported in Table 6. Additional models were conducted to examine interaction effects of HIV status and plasma AFB_1_-lys concentration on HPV 16 and HPV 18 detections, and no significant interaction effects were found. Data of these models are not shown here.

**Table 6:** Multivariable ordinal logistic regression models of persistent HPV 16 and HPV18 detection (persistent detection vs. incidence detection vs. no detection) with demographic and behavioral factors, HIV status, and plasma aflatoxin concentration based on 114 women

Variable	HPV 16		HPV 18	
	OR (95% CI)	*p*-value	OR (95% CI)	*p*-value
Age (years)	1.05 (0.92–1.19)	0.491	1.07 (0.95–1.20)	0.258
Years of education	1.00 (0.81–1.24)	0.995	0.96 (0.79–1.16)	0.665
Married or living with a partner	0.25 (0.04–1.37)	0.110	0.79 (0.18–3.50)	0.759
Home ownership	1.63 (0.21–12.70)	0.642	2.50 (0.46–13.52)	0.287
Travel to health clinic > 30 min	0.55 (0.09–3.40)	0.518	1.55 (0.28–8.63)	0.615
Life-time sex partners	1.12 (0.84–1.49)	0.446	0.93 (0.68–1.27)	0.641
Age of sex debut	1.12 (0.78–1.62)	0.546	0.91 (0.64–1.29)	0.590
HIV-infected	2.16 (0.33–14.00)	0.421	0.40 (0.07–2.26)	0.300
Plasma aflatoxin concentration (pg/uL)	3.16 (1.59–6.26)	0.001	2.06 (1.09–3.90)	0.025

## Discussion

Exposure to dietary aflatoxins is common in sub-Saharan Africa, the main cause being consumption of contaminated corn, the major caloric source, especially for low-income families [47, 48, 49]. In two previous studies conducted by our group, AFB_1_-lys exposure was found to be associated with increased detection and persistence of certain HR-HPV types in Kenyan women who were not living with HIV [35, 36]. In the current study of Ugandan women, plasma AFB_1_-lys concentrations varied from 0.200 to 4.812 pg/µL, a 24-fold range; levels of AFB_1_-lys concentration were similar between WLWH and those not living with HIV. For all women, a higher plasma AFB_1_-lys concentration was associated with a higher risk of persistent detection of HPV types 16 and 18, the two HPV types responsible for nearly 80% of all cervical cancer cases worldwide; this association was independent of HIV status. To our knowledge, no other studies have examined AFB_1_ exposure with association to HR-HPV detection in Ugandan women. We found that all 114 women in the study had detectable plasma AFB_1_-lys at two time points, one year apart. Our finding of high prevalence of AFB_1_ exposure is consistent with those reported from previous studies conducted in Uganda by other groups. One example is a study conducted by Asiki et al.; 100 adults were included, and all 100 blood specimens were positive for aflatoxin biomarkers; those people living near trading centers had higher levels of detectable aflatoxin compared to their counterparts living in rural settings [60]. Kang et al., examined aflatoxin exposure in Southwestern Uganda; greater than 90% of participants were positive [24].

The study presented here revealed an association between AFB_1_ exposure and HR-HPV detection and persistence, the major risk factor for cervical cancer. The implications of this association need further exploration, as the exact mechanism(s) of how aflatoxins alter the immune system are not fully understood [50]. Aflatoxins, being potent carcinogens, may have a direct effect on cervical mucosal tissues, as these compounds have been detected in cervical secretions [51]. Aflatoxins may possibly alter cervical cells to facilitate integration of HR-HPV, as aflatoxins are known to alter DNA integrity [52, 53, 54, 55]. Thus, it is possible that HR-HPV types and dietary aflatoxins act synergistically to increase the risk of cervical cancer, but further studies are needed to explore this possibility.

If long term exposure to aflatoxins has a causative role in persistence of HR-HPV, then millions of exposed women in sub-Saharan Africa are at increased risk of cervical cancer, emphasizing the importance of the goals set by the World Health Organization that include vaccination of young girls and adolescent women against HR-HPV at an age before HPV infection occurs, effective screening of adult women for pre-cancerous lesions of the cervix, and effective treatment of these pre-cancerous lesions [56]. In addition, it is important to develop effective strategies to reduce dietary aflatoxin exposure that may begin at a very young age [49, 57, 58, 59]. Some of these measures include biocontrol with atoxigenic Aspergillus species, genetic manipulation of host plants to increase mold resistance, and farming management methods to reduce mold growth.

Limitations of the present study include a modest sample size. However, a strength of this study is its longitudinal nature; we utilized three measurements of HPV (Enrollment, Month 12, and Month 24) and two measures of plasma AFB_1_ concentration per participant in GEE regression models that enhanced the power of the analysis. Additionally, the association of plasma AFB_1_ concentration and persistent detection of HPV 16 and HPV 18 were strong with relatively small 95% confidence intervals. A second limitation is how “incident detections” were defined, as these could represent reactivation of a latent infection or the end of a persistent infection [60]. Another potential limitation is that dietary factors were not included as a potential confounder in our data analysis. Malnourishment could possibly contribute to suppressed immunity and render such women even more susceptible to the toxic effects of aflatoxins, and thus, more prone to persistent HPV infection [47, 61]. The results of our study may be subject to model overfitting due to a relatively large number of covariates included in a specific model. However, this is unlikely because all covariates included in the constructed models were selected in terms of the findings of prior studies and biological relevance of the chosen variables.

In summary, in this longitudinal study of Ugandan women, every participant had AFB_1_-lys detected in plasma samples at study enrollment and one year later. The plasma concentration of AFB_1_-lys varied widely in the 114 women, and did not differ based on age, HIV status and other variables. A higher plasma AFB_1_-lys concentration was associated with an increased risk of detection and persistence of HPV types 16 and 18, the two oncogenic types most associated with cervical cancer in sub-Saharan Africa and throughout the world. Further studies are needed to determine how aflatoxins increase the risk of oncogenic HPV persistence, and how the effects of these environmental hazards can be mitigated.

## Data Availability

Data is provided within the manuscript.
